# Voltammetric Behavior of o-Nitrophenol and Damage to DNA

**DOI:** 10.3390/ijms9030316

**Published:** 2008-03-12

**Authors:** Da-Peng Zhang, Wei-Li Wu, Hai-Yan Long, Yun-Chun Liu, Zhou-Sheng Yang

**Affiliations:** College of Chemistry and Materials Science, Anhui Key Laboratory of Chemo-Biosensing, Anhui Normal University, Wuhu 241000, P.R. China

**Keywords:** *o*-Nitrophenol, DNA-modified GCE, DNA damage, Electrochemical reduction

## Abstract

The electrochemical behavior of *o*-nitrophenol was studied in detail with a glassy carbon electrode (GCE). The dependence of peak potential on pH indicated that equivalent electrons and protons were involved in the process of *o*-nitrophenol reduction. The interaction of *o*-nitrophenol with calf thymus DNA was investigated by adding DNA to the *o*-nitrophenol solution and by immobilizing DNA on GCE, respectively. The peak current decrement and peak potential shift in presence of DNA indicated that o-nitrophenol could interact with DNA. The result was demonstrated that the in situ DNA damage was detected by differential pulse voltammetry after the *o*-nitrophenol was electrochemically reduced.

## 1. Introduction

Nitrophenols are important and versatile organic compounds in industrial, agricultural and defense applications. They are frequently used as intermediates in the manufacture of explosives, pharmaceuticals, pesticides, pigments, dyes, rubber chemicals and so on [1]. They are produced by microbial hydrolysis of several organophosphorous pesticides, such as parathion [2, 3] or by photodegradation of pesticides that contain the nitrophenol moiety [4, 5]. Nitrophenols also result from natural processes in the biosphere and are now common pollutants in several ecosystems in developed countries [6]. The compounds *o*-nitrophenol, *p*-nitrophenol and 2,4-dinitrophenol are listed on the United State Environmental Protection Agency's (USEPA's) “Priority Pollutants List” [7]. Therefore, the monitoring of *o*-nitrophenol is essential for environmental pollution control.

*o*-Nitrophenol poses significant health risks since it is highly toxic to mammals, microorganisms and anaerobic bacteria. Its toxicity is thought to be due to the nitro group being easily reduced by enzymes to a nitro anion radical, nitroso and hydroxylamine derivatives [1, 8, 9]. These derivatives are responsible for the cytotoxic, mutagenic and carcinogenic action of nitro compounds [10, 11]. Experiments demonstrated that the nitro group reduction products react with nucleic acids *in vitro* and it is believed that DNA is the main target *in vivo* [12]. It was reported that the nitro anion radical is the species principally responsible for DNA damage, oxidizing the DNA double helix and liberating thymidine phosphate [13, 14]. The damage to DNA depends on the stability of the intermediate product formed on nitro reduction. Electrochemical methods have been used to study the reduction and mechanism of action of nitro compounds as an antimicrobial agent [15].

At present, there is a wealth of quantitative information on nitrophenol toxicity in anaerobic treatment systems. More information is required to determine the mechanism of toxicity of these organic compounds [4]. Some heterocyclic nitro compounds, such as metronidazole and benznidazole have been studied by DNA-biosensor [16, 17]. So an electrochemical DNA-modified GCE, prepared by immobilizing dsDNA on a GCE surface, is used to investigate possible DNA damage caused by *o*-nitrophenol. The electrochemical DNA-biosensor enables us to evaluate and predict DNA interaction and damage by health hazardous compounds, based on their binding to nucleic acid, exploring the use of voltammetric techniques for generation of reactive intermediates, which react with DNA *in situ* [18, 19]. The occurrence of damage to dsDNA can be detected by monitoring the oxidation current of the purine bases in DNA molecules. The interpretation of electrochemical data probably makes for elucidation the mechanism of DNA damage [20].

In this paper, the electrochemical behavior of *o*-nitrophenol on GCE was explored using cyclic voltammetry and differential pulse voltammetry. The redox mechanism of *o*-nitrophenol on GCE was discussed. The interaction of *o*-nitrophenol with DNA was investigated with immobilizing DNA on glassy carbon electrode and with adding DNA to solution. The change of electrochemical parameters indicated the *o*-nitrophenol could interact with DNA. Moreover, the oxidative damage to DNA was detected by obtaining the oxidative signal of the purine base in DNA molecules. The possible DNA damage mechanism was proposed.

## 2. Results and discussion

### 2.1. Electrochemical behavior of o-nitrophenol

**Figure 1.**
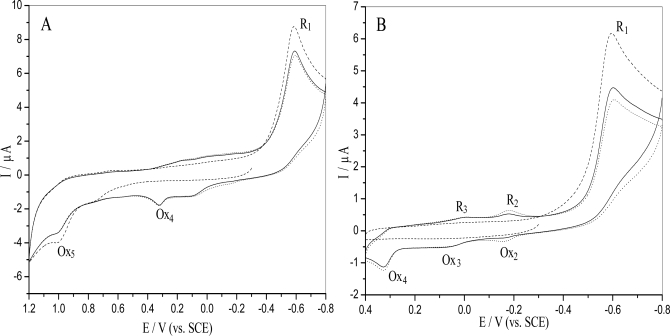
Cyclic voltammograms of 5.0×10^−5^ M o-nitrophenol in 0.10 M pH 4.5 acetate buffer at a bare GCE under N_2_, (---) first cycle; (—) second cycle; (···) third cycle. Scan rate: 50 mV/s, potential rang from (**A**) − 0.8 V to + 1.2 V, E_i_ = − 0.30 V and (**B**) − 0.8 V to + 0.4 V, E_i_ = − 0.30 V.

The reduction of nitrobenzenes is a complex process, especially for those compunds bearing hydroxyl or amine groups in the *ortho*- or *para*- positions [21]. The electrochemical behavior of *o*-nitrophenol on GCE was explored with cyclic voltammetry. Figure 1 showed the cyclic voltammograms of 5.0×10^−5^ M *o*-nitrophenol in 0.10 M pH 4.5 acetate buffer under different electrochemical conditions. Figure 1A was obtained with cycling between − 0.80 V and + 1.20 V, initial potential − 0.30V. A very large irreversible cathodic peak (R_1_) and an irreversible anodic peak (Ox_5_) appeared at around E_pc_ = − 0.602 V and E_pa_ = + 0.998 V in the first cycle, respectively. The R_1_ peak corresponded to a four electrons / four protons reaction, forming hydroxylamine [17, 22], typical of a nitro reduction in acidic aqueous solution, and decreased in subsequent cycles. Peak Ox_5_ at E_pa_= + 0.998 V, was related to the irreversible oxidation of the phenolic group and also decreased in following cycles due to the phenol polymerization at the electrode surface [18]. It was interesting to observe another irreversible anodic peak (Ox_4_) at E_pa_ = + 0.335 V in the second and third cycle, which probably corresponded to condensation reactions expected concerning the oxidation of the reduced intermediates formed, generating electroactive azoxy derivatives [17, 23, 24].

However, two reversible waves could be distinguished if the potential ranged from − 0.80 V to + 0.40 V, with initial potential at − 0.30 V from negative to positive potentials scan (see Figure 1B). On the second and subsequent cycles, the voltammogram was characterized with the one reversible wave centered at − 0.173 V (Ox_2_/R_2_) and the other reversible peak centered at + 0.041 V (Ox_3_/R_3_). The irreversible reduction wave R_1_, corresponding to a four electrons and four protons typical nitro reduction in weak acid aqueous solution, was present in the first scan, but the reversible wave Ox_2_/R_2_ corresponding to nitroso/hydroxylamine derivative, occurred and slowly increased in the second and the following scan, as predicted. It was similar to the behavior of this compound observed at a hanging mercury drop electrode [15, 21]. The latter reversible peak Ox_3_/R_3_ possibly corresponded to the pair azoxy/azo.

**Figure 2.**
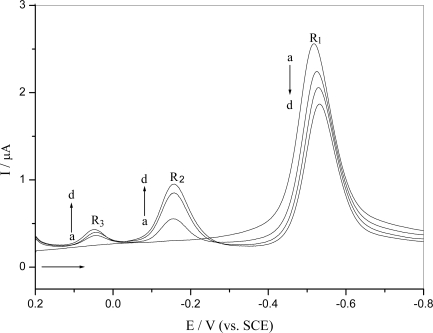
Repetitive differential pulse voltammograms of reduction of 5.0×10^−5^ M o-nitrophenol in 0.10 M pH 4.5 acetate buffer at a bare GCE under N_2_: (a) the first scan, (b) the second scan, (c) the six scan and (d) the fifteenth scan

Repetitive differential pulse voltammograms for reduction of *o*-nitrophenol is shown in Figure 2. The R_2_ and R_3_ wave only appeared in the second and following cycles after the initial *o*-nitrophenol reduction, which was the same with what observed in the cyclic voltammogram. The reduction peak R_1_ corresponding to the nitro group was decreased, while the reduction peak corresponding to the nitroso derivative R_2_ and azoxy derivative R_3_ were increased in subsequent scans. This behavior could be explained by reduction of *o*-nitrophenol leading to its consumption on the electrode surface and electrogenerating a hydroxylamine derivative that adsorbed onto the electrode surface and was oxidized to nitroso and other derivatives.

**Figure 3.**
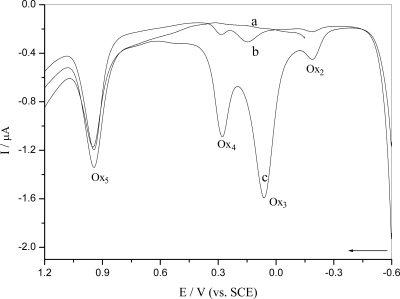
Differential pulse voltammograms of 5.0×10^−5^ M *o*-nitrophenol in 0.10 M pH 4.5 acetate buffer at a bare GCE under N_2_: (a) E_i_ = − 0.15 V, (b) E_i_ = − 0.6 V and (c) E_i_ = − 0.60 V after E_d_ = − 0.6 V for t_d_ = 2 minutes

Figure 3 shows the differential pulse voltammogram for oxidation of *o*-nitrophenol starting at different potentials and deposition at a constant potential. It was confirmed that peak Ox_5_ was due to the irreversible oxidation of the phenolic group, as only this group is able to undergo oxidation in *o*-nitrophenol (Figure 3, curve a), and that Ox_2_, Ox_3_ and Ox_4_ were generated only after *o*-nitrophenol was reduced at − 0.60 V (Figure 3, curve b and c). It caused an increase in the peak Ox_2_, Ox_3_ and Ox_4_ after applying a potential of − 0.6 V for 2 minters (Figure 3, curve c), which produced abundant hydroxylamine in this process.

In a similar way to what was observed for nitroaromatic compounds [16, 17], *o*-nitrophenol reduction was also pH dependent in weak acidic medium. The potential of reduction peak shifted to negative values with increasing pH. The variation of E_pc_ for the reduction of *o*-nitrophenol with pH showed the slop of 52 mV/pH (data not shown). This behavior indicated the same number of electrons and protons participated in the reduction of *o*-nitrophenol.

### 2.2. o-Nitrophenol-DNA interaction

#### 2.2.1. Interaction of o-nitrophenol with DNA in solution

**Figure 4.**
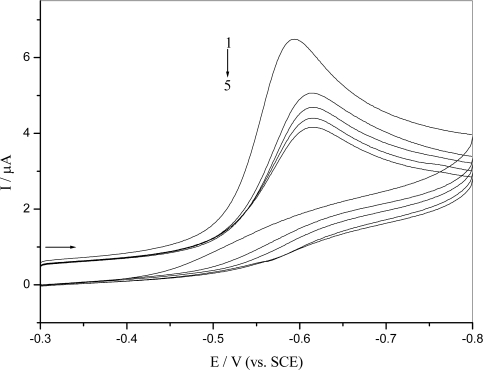
Cyclic voltammograms of 5.0×10^−5^ M *o*-nitrophenol at GCE in 0.10 M pH 4.5 acetate buffer in absence (1) and presence of 28 ug/mL (2), 56 ug/mL (3), 84 ug/mL (4), 112 ug/mL (5) dsDNA. Scan rate: 50mV/s.

The above results indicate that *o*-nitrophenol could exhibit fine electrochemical response, so the interaction of *o*-nitrophenol with DNA could be studied by electrochemical methods [25–27]. The typical cyclic voltammograms of 5.0×10^−5^ M *o*-nitrophenol on glassy carbon electrode in absence and presence of dsDNA in 0.10 M pH 4.5 acetate buffer are shown in Figure 4. A decrease in peak current and shift of peak potentials to more negative values when dsDNA was added to a solution could be easily observed. The decrease of peak current was due to the diffusion of *o*-nitrophenol-DNA complex decrement, not due to the increased viscosity of the solution or the blockage of the electrode surface by an adsorbed layer of DNA. Special cyclic voltammograms were recorded for 0.5mM K_4_[Fe(CN)_6_], which cannot interact with DNA at GCE in the absence and presence of DNA. The peak current of K_4_[Fe(CN)_6_] was not nearly affected and the peak potential didn't shift any after addition of DNA. Thus, there was hardly impacted on the diffusion from the changed viscosity of DNA addition, and no significant obstruction on the GCE surface from DNA adsorption. A great decrease in peak current and a shift in potential in above CV experiments was attributed to *o*-nitrophenol bound to the bulky, slowly diffusing DNA to form *o*-nitrophenol-DNA adduct, which resulted in a considerable decrease in the apparent diffusion coefficient.

#### 2.2.2 Interaction of o-nitrophenol with DNA immobilized at GCE surface

**Figure 5.**
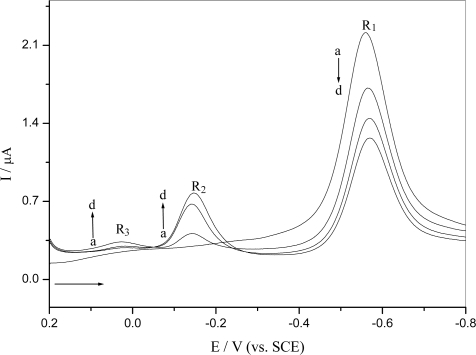
Repetitive differential pulse voltammograms of reduction of 5.0×10^−5^ M *o*-nitrophenol in 0.10 M pH 4.5 acetate buffer at the DNA-modified GCE under N_2_: (a) the first scan, (b) the second scan, (c) the six scan and (d) the fifteenth scan.

Figure 5 shows the repetitive differential pulse voltammogram of *o*-nitrophenol electrochemical reduction using a DNA-modified GCE. It could be found that its reduction behavior on a DNA-modified GCE was similar to what happened at the bare GCE (Figure 2), but the potential shifted to negative value and the peak current decreased about 12% compared to their first scan. It indicated that pre-concentration of hydroxylamine onto the DNA matrix, denoted by the increase of the reduction peak for the nitroso R_2_ and azoxy R_3_ derivative with successive scans. Thus the DNA gel on the electrode surface could accumulate the analyte on it [18].

### 2.3 DNA damage

**Figure 6.**
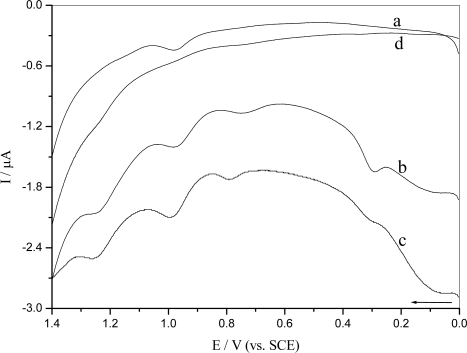
Differential pulse voltammograms using DNA-modified GCE in 0.10 M pH 4.5 acetate buffer containing 1.0×10^−5^ M *o*-nitrophenol before (a) and after reduction (b) by successive differential pulse voltammetry scans (10) from 0 to − 0.8 V and (c) at constant potential E_d_ = − 0.60 V for t_d_ = 2 minutes. (d) Differential pulse voltammogram obtained with the DNA-modified GCE in 0.10 M pH 4.5 acetate buffer.

In order to investigate that electrochemical reduction of *o*-nitrophenol generates short-lived radical that interacts with DNA causing damage, differential pulse voltammetry was applied to detect DNA damage using a DNA-modified GCE. The blank experiment, i.e. oxidation at DNA-modified GCE without *o*-nitrophenol (Figure 6d), was performed in buffer solution to test the above hypothesis. There were almost no oxidation peaks of the DNA base, because nucleobases within native DNA are hidden inside the helix and stiffness of the structure keeps them far from the electrode surface [28]. Figure 6a shows the differential pulse voltammetry of *o*-nitrophenol oxidation using DNA-modified GCE. There was only an oxidation peak at + 0.982 V, which corresponded to the *o*-nitrophenol oxidation (Figure 6 curve a). However, the damage to DNA was detected if *o*-nitrophenol was reduced at a constant deposition potential E_d_ = − 0.60 V for 2 min (Figure 6, curve c). The appearance of two oxidative peak was attributed to guanine, E_pa_ = + 0.785 V and adenosine, E_pa_ = + 1.271 V [29]. In addition, the peak current at E_pa_ = + 0.982 V increased. It was interesting to observe DNA damage if the reduction of *o*-nitrophenol was performed in repetitive differential pulse scanning from 0 to − 0.80 V (10 scans) (Figure 6, curve b). This distinctly indicated that *o*-nitrophenol could damage to DNA after the *o*-nitro-phenol underwent reduction process. The radicals generated in reduction process of the *o*-nitrophenol interacted with DNA [22, 30]. The appearance of purine oxidation peak demonstrated clearly that the radicals caused distortion of DNA double helix and exposure of the bases.

### 2.4 Mechanism of the o-nitrophenol redox and damage to dsDNA

The above experimental results provided some evidence that reduction of *o*-nitrophenol was a complex process. Its electrochemically reduction process underwent some intermediate product formation such as nitro radical anion, nitroso, hydroxylamine, etc. The radical formed by reduction of the nitro group could interact with dsDNA and cause dsDNA damage. After the *o*-nitrophenol was reduced, the detected oxidation current signal of the guanine and adenosine nucleobase supplied evidences of DNA damage. According to above experimental results and reference to [31], a possible mechanism of the *o*-nitrophenol redox and damage to dsDNA was proposed as follows:

## 3. Experimental

### 3.1 Chemicals and Apparatus

Calf thymus DNA (sodium salt, type I) was purchased from Sigma and *o*-nitrophenol was received from Shanghai Chemical Reagent Company, respectively. They were used without further purification. Acetate buffer solution of 0.10 M (pH = 4.5) was prepared using analytical grade reagents. Double-distilled water was used throughout. All experiments were performed at room temperature.

Stock solution of 5.0×10^−3^ M *o*-nitrophenol was prepared in a 1:1 mixture of pH = 4.5 0.10 M acetate buffer and ethanol. The stock solution was diluted with 0.10 M acetate buffer according to the demand. The solution was saturated with N_2_ by bubbling high purity nitrogen gas into solution for 15 min before experiment. Nitrogen gas was maintained over the solution by continuing with a flow of the pure gas during the voltammetric experiment.

All voltammetric measurements were carried out using a CHI440A electrochemical analyzer (Shanghai Chenhua Apparatus Corporation, China). The experiments were performed using a 5 mL one-compartment electrochemical cell with a three electrodes system, which was consisted of a glassy carbon electrode or DNA-modified glassy carbon electrode as working electrode, a platinum coil auxiliary electrode and a saturated calomel electrode (SCE) as reference electrode.

### 3.2 The process of preparing DNA-modified glassy carbon electrode

The DNA-modified glassy carbon electrode (Φ = 3 mm) was prepared as follows: It was polished using a piece of 1500 diamond paper, and then polished to mirror smoothness with about 0.05 um alumina water slurry on silk. Afterward, it was washed with absolute alcohol and double-distilled water in an ultrasonic bath to remove the adsorbates on the electrode. After electrode was dried in air, 20 μL of DNA solution (2.0 mg/mL) was dropped onto the surface of the clean glassy carbon electrode and solvent was evaporated at 4 °C for 12 hours. The modified electrode was immersed the double-distilled water for 15 min to remove the unadsorbed DNA before using.

## 4. Conclusions

This work showed that the electrochemical behavoiur of *o*-nitrophenol at a GCE was complex. The interaction of *o*-nitrophenol-DNA was studied by using the dsDNA film-modified GCE. The result suggested that *o*-nitrophenol caused DNA damage *in situ* after it was reduced. These results also contributed in understanding the possible mechanism of DNA damage.

## Figures and Tables

**Scheme 1.**
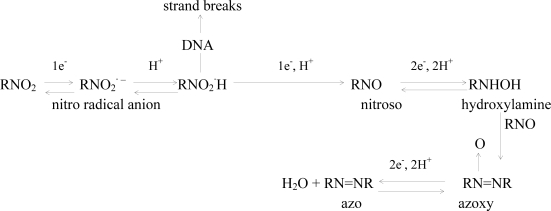
Reduvtive process of o-nitrophenol and feasible damage to DNA (R = phenol group). Adapted from the reference [31].
